# Causality between Ankylosing Spondylitis and osteoarthritis in European ancestry: a bidirectional Mendelian randomization study

**DOI:** 10.3389/fimmu.2024.1297454

**Published:** 2024-02-06

**Authors:** Yangguang Lu, Di Lu, Hongzhi Zhang, Haoyang Li, Bohuai Yu, Yige Zhang, Hantao Hu, Hongfeng Sheng

**Affiliations:** ^1^ The First School of Medicine, School of Information and Engineering, Wenzhou Medical University, Wenzhou, China; ^2^ Department of Orthopedics, Tongde Hospital of Zhejiang Province, Hangzhou, China

**Keywords:** Ankylosing Spondylitis, osteoarthritis, Mendelian randomization, genetic analyses, orthopedics, inflammation

## Abstract

**Objective:**

To explore the bidirectional causal relationship between Ankylosing Spondylitis (AS) and Osteoarthritis (OA) at the genetic level within the European ancestry.

**Methods:**

We implemented a series of quality control steps to select instrumental variables (IVs) related to the exposure. We conducted two-sample Mendelian randomization (MR) using the inverse-variance weighted method as the primary approach. We adjusted significance levels using Bonferroni correction, assessed heterogeneity using Cochrane’s Q test. Sensitivity analysis was conducted through leave-one-out method. Additionally, external datasets and relaxed IV selection criteria were employed, and multivariate MR analyses were performed for validation purposes. Finally, Bayesian colocalization (COLOC) analysis identified common genes, validating the MR results.

**Results:**

The investigation focused on the correlation between OA and AS in knee, hip, and hand joints. MR results revealed that individuals with AS exhibit a decreased risk of knee OA (OR = 0.9882, 95% CI: 0.9804-0.9962) but no significant increase in the risk of hip OA (OR = 0.9901, 95% CI: 0.9786-1.0018). Conversely, AS emerged as a risk factor for hand OA (OR = 1.0026, 95% CI: 1.0015-1.0036). In reverse-direction MR analysis, OA did not significantly influence the occurrence of AS. Importantly, minimal heterogeneity was observed in our MR analysis results (*p* > 0.05), and the robustness of these findings was confirmed through sensitivity analysis and multivariate MR analysis. COLOC analysis identified four colocalized variants for AS and hand OA (rs74707996, rs75240935, rs181468789, and rs748670681).

**Conclusion:**

In European population, individuals with AS have a relatively lower risk of knee OA, whereas AS serves as a risk factor for hand OA. However, no significant causal relationship was found between AS and hip OA. Additionally, it offers novel insights into genetic research on AS and OA.

## 1 Introduction

Osteoarthritis (OA) is a prevalent chronic degenerative joint disease with an unclear etiology, exerting a substantial global impact (1, 2), and serving as a significant contributor to disability (3). It predominantly affects the hip and knee joints, characterized by progressive deterioration of articular cartilage, subchondral bone remodeling, chondrocyte hypertrophy, and synovial inflammation (4, 5). This condition imposes a considerable burden on healthcare resources, affecting over 500 million people worldwide, which is approximately 7% of the world’s population (6). The incidence of OA is rising due to aging demographics and the prevalence of obesity (7). Additionally, the financial burden on OA patients is increasing (8), particularly in Western nations, where it accounts for an estimated 1% to 2.5% of the gross domestic product (9). For individuals with mild to moderate OA, conservative treatments are available, but their specific efficacy remains contentious (10). In advanced cases, total joint replacement surgery is a mature option, yet not all patients are suitable candidates, and post-operative complications can occur (11). OA is a complex condition influenced by multiple factors, including trauma, metabolism, biological stress, and genetic susceptibility (12, 13). Despite ongoing research, the precise mechanisms underlying OA pathogenesis remain incompletely elucidated. Therefore, a better understanding of its etiology and early prevention and treatment strategies is crucial.

Ankylosing spondylitis (AS) is an immune-mediated inflammatory arthritis characterized by a strong genetic predisposition, primarily affecting the axial skeleton, leading to sacroiliac joint damage, fusion, vertebral ankylosis, and the classic bamboo spine appearance, along with functional impairment and a reduced quality of life (14–16). AS predominantly affects young males, with about three-quarters of patients experiencing initial symptoms before the age of 30 (17). Research indicates that the highest risk of AS diagnosis occurs between the ages of 30 and 40, with common symptoms manifesting as non-specific back pain in the lower back and buttocks (18). Moreover, AS can also induce peripheral symptoms in the knees, hands, and feet, resembling rheumatoid arthritis (16). This can lead to misdiagnosis and delayed appropriate treatment, resulting in a frequently delayed diagnosis by 5 to 10 years (18).

Previous research has indicated that inflammation in AS may accumulate, potentially increasing the risk of secondary OA (19). Notably, the hip and knee joints, being the largest weight-bearing joints in the human body, are commonly affected by AS among peripheral joint. A cohort study conducted in Taiwan found a notably higher incidence of OA in male AS patients, who also showed a higher frequency of undergoing total hip replacement surgery (THRS) and total knee replacement surgery (TKRS) (19). A possible explanation for this phenomenon is that peripheral joint inflammation in AS results from the migration of HLA-B27 positive immune cells into the joints, triggering a local inflammatory response (20). Understanding the intricate relationship between AS and OA could pave the way for more precise prevention and treatment strategies. Nevertheless, the exact association between AS and OA remains unclear, necessitating further evidence for substantiation.

Mendelian randomization (MR) studies make use of genetic variations, such as single nucleotide polymorphisms (SNPs), which naturally occur during meiosis. This approach minimizes confounding factors and reverse causation bias in epidemiological research, as genetic variations precede disease onset (21). Consequently, MR studies enable the exploration of bidirectional causal relationships between OA and AS. In light of this, we conducted a bidirectional two-sample MR study to investigate the potential causal link between AS and OA at different anatomical sites.

## 2 Materials and methods

### 2.1 Study design

This study adhered to the Strengthening the Reporting of Observational Studies in Epidemiology Using Mendelian Randomization (STROBE-MR) guidelines (21). We utilized summary data from genome-wide association studies (GWAS) on AS and OA to select suitable NPs as instrumental variables (IVs) for MR analysis. Our objective was to investigate bidirectional causal relationships between AS and OA. The application of IVs in MR analysis relies on satisfying three critical assumptions: (i) the chosen IVs exhibit a robust association with the exposure of interest, (ii) there is no confounding of the IVs with factors influencing the outcome apart from the exposure, and (iii) the selected IVs solely influence the outcome through the exposure (22). Since all data were derived from previously published studies and public databases, no additional ethical approval was required.

### 2.2 GWAS summary data for AS

Genetic data for AS were obtained from the FinnGen consortium (https://r9.finngen.fi/, last accessed on September 2, 2023). The study cohort consisted of individuals of European descent who provided informed consent. The FinnGen research project integrated genetic data related to disease endpoints from the Finnish Biobank and Finnish Health Registry, encompassing a total of 2,860 AS patients and 270,964 healthy controls. Case identification was based on codes from the Tenth Revision of the International Classification of Diseases (ICD-10). Detailed information regarding participant characteristics, genotyping, imputation, and quality control can be found on the FinnGen website (https://finngen.gitbook).

To address potential population-specific limitations in the AS data from the FinnGen consortium and further validate our findings, we conducted a replication analysis using summary data from a genome-wide association study for AS published by the International Genetics of Ankylosing Spondylitis (IGAS) consortium (23) in 2013. This dataset consisted of European population cohorts, including 9,069 AS cases and 13,578 healthy controls, all of whom provided informed consent. Case definitions were based on ICD-10 codes. A comprehensive description of the study procedures is available in previously published research (23).

### 2.3 GWAS summary data for OA

We obtained summary genetic data for OA from the largest sample GWAS meta-analysis of OA, conducted by Dr. Cindy G. Boer et al. (24) in 2021. This extensive study incorporated data from 13 international OA cohorts. Our focus was on extracting relevant GWAS information related to knee OA, hip OA, and hand OA. The dataset included 62,497 cases and 333,557 healthy controls for knee OA, 36,445 cases and 316,943 healthy controls for hip OA, and 20,901 cases and 282,881 healthy controls for hand OA. The majority of participants were of European descent and provided informed consent. Case definitions were based on ICD-10 codes. Comprehensive research procedure details are available in the published study (24). Importantly, there was no overlap in populations between our study and the two cohorts related to AS.

### 2.4 Instrumental variable selection

To ensure the robustness and reliability of our MR analysis, we implemented a rigorous selection process for instrumental variables (IVs). Firstly, we identified SNPs closely associated with the exposure (*p* < 5 × 10^−8^). Secondly, we eliminated linkage disequilibrium (LD) between SNPs, as strong LD can introduce bias (r² < 0.001, clumping distance = 10,000 kb). In cases of LD genetic variants, we chose the variant with the lowest *p*-value associated with the exposure. Subsequently, we filtered out SNPs with an F > 10 to ensure a strong correlation between IVs and the exposure, excluding weak instrumental variables. Finally, we manually screened and removed SNPs associated with confounding factors and OA outcomes using the PhenoScanner database (http://www.phenoscanner.medschl.cam.ac.uk/, accessed on September 3, 2023). If multiple SNPs were missing in GWAS results, we searched for proxies using the LDlink online platform (https://ldlink.nci.nih.gov/, accessed on September 3, 2023). Any replaced SNPs were excluded from the final MR analysis.

We identified 10 SNPs associated with AS in the FinnGen consortium dataset. One SNP (rs181316459) was excluded due to its status as a proxy SNP, and no other SNPs were proxied (**Table 1**). To enhance the sensitivity of positive results, a sensitivity analysis was conducted on this dataset by relaxing the instrumental variables (IVs) selection criteria (*p* < 1 × 10^−6^, r² < 0.001, F > 10). This analysis identified 41 additional SNPs. In the IGBS consortium dataset, we identified 24 SNPs associated with AS (23). One SNP (rs130075) lacked the necessary information for MR testing, and one SNP (rs2517655) was missing from the results. Since the SNP missing rate fell within an acceptable range and to maintain population stability, proxy SNPs were not sought. Furthermore, in the dataset from Dr. Boer et al. (24), we identified 24 SNPs associated with knee OA, 33 SNPs with Hip OA, and 8 SNPs with hand OA. None of these were proxied.

**Table 1:** Extraction of instrumental SNPs for Mendelian randomization analysis from the GWAS study of ankylosing spondylitis.

SNP	Position	A1	A2	EAF	Beta	SE	*p*-value
rs10456271	6:23108189	T	C	0.0118	0.4553	0.0676	1.61E-11
rs10807943	7:5301033	C	T	0.1028	-0.3737	0.0494	3.77E-14
rs114799031	6:28185504	T	A	0.9747	1.2742	0.0364	8.28E-269
rs13192159	6:25583940	T	C	0.1809	0.5337	0.0315	2.50E-64
rs142695953	7:5804237	A	C	0.1391	0.2147	0.0323	7.38E-11
rs2032890	5:96785448	C	A	0.6996	-0.2432	0.0312	6.97E-15
rs6759003	2:62332070	C	T	0.3609	-0.1880	0.0277	1.12E-11
rs72749142	1:200982179	A	G	0.1677	-0.2353	0.0411	1.04E-08
rs78724843	6:35948243	A	C	0.0334	0.6199	0.0537	9.54E-31
rs9461388	6:27522650	C	T	0.9705	-0.5790	0.0957	1.44E-09

A1, Effect allele; A2, Other allele; EAF, Effect allele frequency; SE, Standard error.

### 2.5 Mendelian Randomization analysis

**Figure 1:**
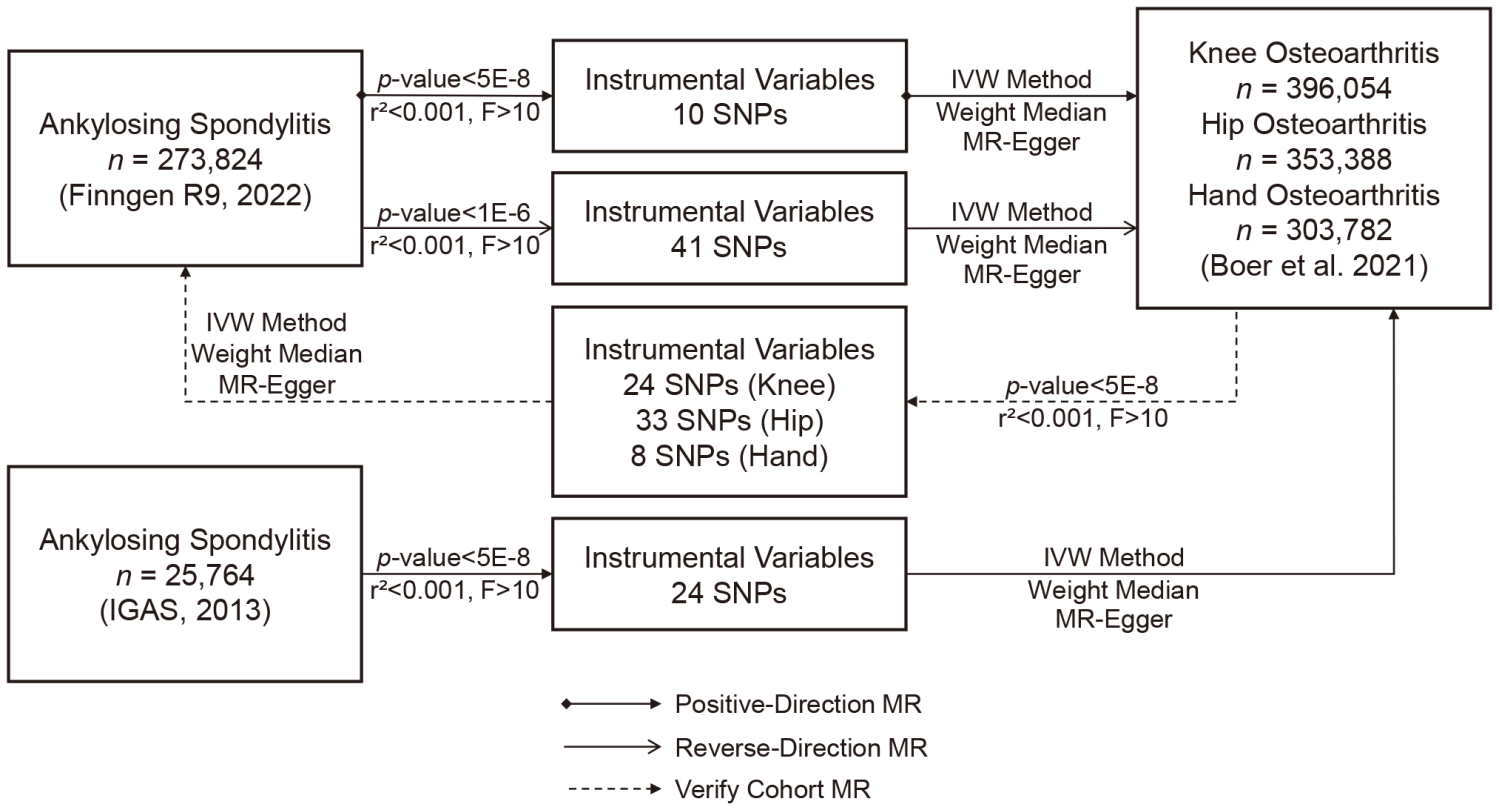
Flowchart of the study design overview.

Our primary analytical method for assessing the correlation between exposure and outcomes was Inverse Variance Weighting (IVW). This method provides accurate estimates when all IVs are valid (25). In addition, we employed MR-Egger regression and the weighted median as supplementary analytical methods. MR-Egger regression is capable of detecting and adjusting for pleiotropy, although it tends to produce estimates with lower precision (22). The weighted median approach provides accurate estimates, assuming that at least 50% of IVs are valid (26). We presented our results in the form of odds ratios (ORs) and 95% confidence intervals (CIs). To gauge heterogeneity, we utilized Cochrane’s Q test and quantified it with I². Heterogeneity was considered absent when I² was below 25% and mild when it was below 50%. We assessed potential horizontal pleiotropy through the intercept of MR-Egger regression and MR pleiotropy residual sum and outlier (MR-PRESSO) (22, 27). To ensure the robustness of our results, we conducted a sensitivity analysis using the leave-one-out method to identify SNPs that might exert potential influence. We also assessed the robustness of positive findings by either relaxing the IV selection criteria (*p* < 1 × 10^−6^, r² < 0.001, F > 10) to obtain more IVs or performing MR analysis using another GWAS cohort for result validation (**Figure 1**). Moreover, given previous research highlighting the genetic correlation between OA occurrence and body mass index (BMI) (28), we conducted an additional multivariate MR analysis using summary data from the FinnGen R9, where BMI served as the exposure variable. This supplementary analysis was undertaken to disentangle the potential influence of BMI, providing a more comprehensive understanding of its impact on the observed associations.

Considering that our analyses encompassed three different OA sites, we took a conservative approach to address multiple testing. Specifically, we applied Bonferroni correction to the significance level, setting a threshold at 0.05 divided by 3 (i.e., 0.0167). Consequently, *p*-values less than 0.05 but above the Bonferroni-corrected significance threshold were deemed as potential evidence of association (29). For all other tests, *p*-values less than 0.05 were considered statistically significant. We conducted the statistical analyses using R version 4.3.2 and the “TwoSampleMR” package.

### 2.6 Colocalization analysis

To further validate the results of MR and explore the genetic connections between AS and OA, we conducted Bayesian colocalization (COLOC) analysis based on GWAS summary statistics of AS and OA. The data were sourced from FinnGen R9 and the study conducted by Dr. Boer et al. (24). COLOC analysis, a statistical method rooted in GWAS data, employs single-variant summary statistics to assess whether two independently associated genetically correlated traits share a common genetic locus. In this analysis, we assigned a prior probability of 1 × 10^−6^ to the random variable having a causal relationship with both GWAS datasets. This value indicates a sufficiently high posterior probability (PP4 > 0.8) for a single shared variant between AS and OA (30). We conducted this statistical analyses using R version 4.3.2 and the “coloc” package.

## 3 Results

### 3.1 MR analysis

**Figure 2:**
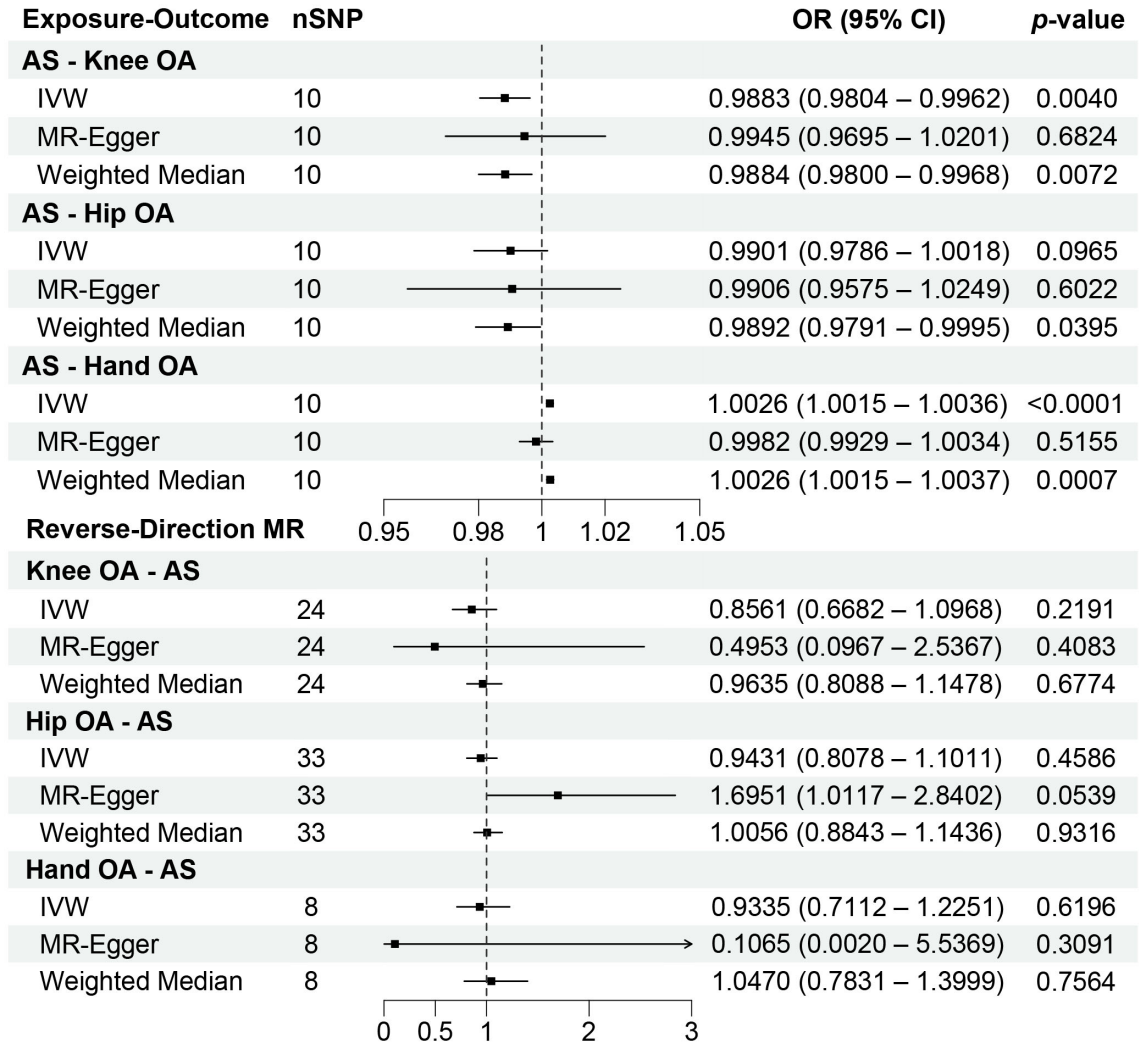
Forest plots of the results on two-sample bidirectional Mendelian randomization analysis. OR, Odds ratio.

**Figure 3:**
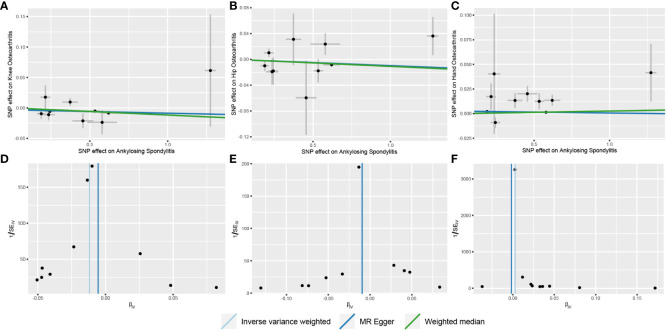
Scatter and funnel plots of the results of Mendelian randomization with AS as an exposure factor. **(A)** Scatterplot with knee OA as the outcome; **(B)** Scatterplot with hip OA as the outcome; **(C)** Scatterplot with hand OA as the outcome; **(D)** Funnel plot with knee OA as the outcome; **(E)** Funnel plot with hip OA as the outcome; **(F)** Funnel plot with hand OA as the outcome.

Our analysis using the random-effects IVW model yielded insightful findings. Individuals with AS exhibited a relatively lower risk of knee OA (OR = 0.9883, 95% CI: 0.9804-0.9962, *p* = 0.0040). However, AS did not significantly affect the occurrence of hip OA (OR = 0.9901, 95% CI: 0.9786-1.0018, *p* = 0.0965). In contrast, AS was identified as a risk factor for Hand OA (OR = 1.0026, 95% CI: 1.0015-1.0036, *p* < 0.0001) (**Figure 2**). When assessing the impact of AS on knee OA, both IVW and Weighted Median models provided consistent results. Although MR-Egger regression did not indicate significant outcomes, a persistent trend toward a lower risk of knee OA in individuals with AS was observed (**Figure 3A**). For the evaluation of AS on hip OA, all three test models yielded similar results (**Figure 3B**). In the assessment of AS on hand OA, the IVW and Weighted Median models produced consistent outcomes, while MR-Egger resulted in nonsignificant results (**Figure 3C**).

The results of Cochrane’s Q test demonstrated no heterogeneity in the MR analysis between AS and knee OA (**Figure 3D**) (*p* = 0.2620, I²= 19.67%). For hip OA (**Figure 3E**), there was only mild heterogeneity (*p* = 0.1180, I² = 36.27%). However, a moderate level of heterogeneity was observed in the MR analysis between AS and hand OA (**Figure 3F**) (*p* = 0.0007, I² = 68.90%). Importantly, both MR Egger intercept tests and MR-PRESSO distortion tests indicated the absence of horizontal pleiotropy between AS and OA at all three sites (*p* > 0.05) (**Table 2**).

**Table 2:** Heterogeneity and horizontal pleiotropy analyses between ankylosing spondylitis and osteoarthritis.

Exposure	Outcome	Heterogeneity				Horizontal Pleiotropy		
		Q	Q df	I²	*p*-value	Egger intercept	SE	*p*-value
AS	Knee OA	11.20	9	19.67	0.2620	-0.0031	0.0061	0.6225
AS	Hip OA	14.12	9	36.27	0.1180	-0.0003	0.0094	0.9751
AS	Hand OA	28.94	9	68.90	0.0007	0.0025	0.0015	0.1358
Knee OA	AS	25.93	23	11.30	0.3042	0.0284	0.0427	0.5133
Hip OA	AS	32.91	32	2.76	0.4224	-0.0453	0.0195	0.0264
Hand OA	AS	6.71	7	4.30	0.4595	0.1710	0.1583	0.3218

SE, Standard error.

### 3.2 Reverse-direction MR analysis

In the reverse-direction MR analysis, the random-effects IVW model revealed that knee OA (OR = 0.8561, 95% CI: 0.6682-1.0968, *p* = 0.2191), hip OA (OR = 0.9431, 95% CI: 0.8078-1.1011, *p* = 0.4586), and hand OA (OR = 0.9335, 95% CI: 0.7112-1.2251, *p* = 0.6196) did not significantly influence the occurrence of AS. In fact, across all MR models, the results consistently yielded nonsignificant statistical outcomes (*p* > 0.0167) (**Figure 2**).

Cochrane’s Q test results indicated the absence of heterogeneity in the MR analysis between OA and AS (knee OA: *p* = 0.3042, I²= 11.30%; hip OA: *p* = 0.4230, I² = 12.86%; hand OA: *p* = 0.4595, I² = 4.30%). While the MR Egger intercept tests showed the absence of horizontal pleiotropy between knee OA, hand OA, and AS (*p* > 0.05), some level of horizontal pleiotropy was observed between Hip OA and AS (*p* = 0.0264) (**Table 2**). Nevertheless, despite this observation, the models continued to yield negative combined results. Additionally, MR-PRESSO distortion tests indicated the absence of outliers related to OA instrumental variables.

### 3.3 Sensitive analysis

The leave-one-out sensitivity analysis demonstrated the robustness of the combined results from the IVW model in all MR analyses. No significant changes were observed upon excluding individual SNPs. For statistically significant findings, we conducted further verification (**Table 3**). We also expanded our analysis using instrumental variables obtained from the relaxed selection criteria, selecting a total of 41 instrumental variables related to AS. The influence of AS on knee OA (OR = 0.9982, 95% CI: 0.9976-0.9987, *p* < 0.0001) and hand OA (OR = 1.0026, 95% CI: 1.0015-1.0038, *p* < 0.0001) remained largely consistent with the previous results. In the validation MR analysis using an alternative cohort to investigate the relationship between AS and knee OA and hand OA, the results confirmed a lower risk of knee OA in individuals with AS (OR = 0.9959, 95% CI: 0.9946-0.9972, *p* < 0.0001). However, the significant association between AS and hand OA disappeared in the validation MR analysis (OR = 1.0102, 95% CI: 0.9998-1.0208, *p* = 0.0556). Considering the differentiated outcomes from the two datasets, we employed a meta-analysis approach to consolidate the corresponding OR values, which consistently indicated that AS serves as a risk factor for hand OA (OR = 1.0027, 95% CI: 1.0016-1.0037, *p* < 0.0001). In the context of the multivariate MR analysis accounting for BMI, we still observed at the genetic level that individuals with AS have a relatively lower risk of knee OA (OR = 0.9954, 95% CI: 0.9919-0.9988, *p* = 0.0082), while concurrently exhibiting an increased risk of hand OA (OR = 1.0028, 95% CI: 1.0020-1.0036, *p* < 0.0001).

**Table 3:** Results of analyses using instrumental variables screened with low constraints, external validation cohorts, or multivariate MR analyses combined with BMI variables.

Outcome	Model 1		Model 2		Model 3	
	OR	*p*-value	OR	*p*-value	OR	*p*-value
Knee OA	0.9982 (0.9976-0.9987)	<0.0001	0.9959 (0.9946-0.9972)	<0.0001	0.9954 (0.9919-0.9988)	0.0082
Hand OA	1.0026 (1.0015-1.0038)	<0.0001	1.0102 (0.9998-1.0208)	0.0556	1.0028 (1.0020-1.0036)	<0.0001

OR, Odds ratio; Model 1, Relaxation of screening conditions for instrumental variables to *p* < 1 × 10^−6^, r² < 0.001, F > 10; Model 2, MR analysis using an external AS validation queue; Model 3, Multivariate MR analysis conducted using BMI as an adjustment variable.

### 3.4 Colocalization between AS and OA

In the pairwise GWAS of AS and hand OA, four loci, namely rs74707996 (PP4 = 0.9999), rs75240935 (PP4 = 0.9999), rs181468789 (PP4 = 0.9484), and rs748670681 (PP4 = 1), were classified as colocalized through COLOC analysis. Notably, except for rs748670681 located on chromosome 7, the remaining three loci are situated on chromosome 6. Furthermore, the nearest genes to rs74707996, rs75240935 and rs181468789 are *NRSN1*, *SLC17A3* and *OR2B6*, but all are at distances more than 100bp, while rs748670681 is located on the gene region of *TNRC18*.

## 4 Discussion

This study marks the pioneering exploration of the bidirectional relationship between AS and OA through Mendelian randomization analysis. Despite our research indicating only a modest genetic association between AS and knee OA as well as hand OA, the observed correlations carry significant *p*-values. Additionally, the COLOC analysis highlights four loci classified as colocalized with AS and hand OA, emphasizing the relevance of these subtle genetic effects. Therefore, the modest genetic associations uncovered at the genetic level should not be overlooked. Our conclusions based on the genetic level that differ from past perceptions also require new attention.

The intricate interplay between AS and OA has long confounded researchers, with limited existing literature and no definitive consensus. Notably, the pathogenesis and pathology of AS and OA are distinct; AS constitutes an autoimmune disease (15), whereas OA involves degenerative cartilage alterations (4). Our research findings significantly deviate from conventional clinical wisdom, particularly the discovery of a relatively lower risk of knee OA in patients with AS. One plausible mechanism could be attributed to individuals with ankylosing spondylitis often exhibiting elevated levels of serum bone formation markers like bone alkaline phosphatase, osteocalcin, and bone-specific alkaline phosphatase (31). These markers reflect the body’s bone metabolism activity and may also correlate with cartilage metabolism (32), with knee joint cartilage metabolism being particularly sensitive to them. Alternatively, from a pharmacological standpoint, AS patients frequently require prolonged usage of non-steroidal anti-inflammatory drugs (NSAIDs) to manage symptoms and impede structural progression (33). NSAIDs not only inhibit prostaglandin E2 (PGE2) synthesis, thus mitigating inflammatory responses, but also obstruct osteoblast differentiation, curtailing new bone formation (34, 35). PGE2 serves as a pivotal mediator that incites degenerative changes in cartilage, prompting chondrocytes to release matrix metalloproteinases (MMPs), ultimately leading to cartilage matrix degradation (36, 37). Consequently, NSAID utilization might contribute to shielding knee joint cartilage from PGE2-induced harm.

It is worth noting that hip joint involvement frequently manifests in AS patients, with clinical hip joint involvement rates ranging from 24% to 36% and radiographic hip joint arthritis rates spanning 9% to 22% (38). Prior investigations have elucidated potential underlying mechanisms, revealing 193 differentially expressed proteins (DEPs) predominantly enriched in functional pathways like the phagosome pathway in ligament-like hip joint samples of AS patients. These pathways might represent pivotal pathogenic routes influencing hip joint engagement in AS patients. Notably, Myeloperoxidase (MPO), a pivotal protein in the phagosome pathway, registers upregulation in the AS group. MPO may potentially spur autoimmune inflammation in hip joints via the phagosome pathway, culminating in hip joint lesions (39). However, our study did not confirm a significant direct genetic association between AS and hip OA. One plausible explanation is that the effect of reducing the risk of developing knee OA in patients with AS may also extend to the hip joint, mitigating the impact of AS on hip joint involvement. Similarly, AS’s risk factor impact on hand OA might originate from analogous mechanisms but exert heightened sensitivity when influencing the hand. Furthermore, the migration of HLA-B27-positive immune cells induced by AS to the hand joints via the bloodstream could potentially incite an inflammatory response (20).

When validating our MR analysis results using data from the IGAS alliance (23) in 2013, a trend of AS causing hand OA persists, but the statistical significance has disappeared. This phenomenon can be attributed to several factors. Innovative treatment strategies and drugs for AS, such as the IL-12/23 inhibitor ustekinumab, the pan-Janus kinase inhibitor tofacitinib, and the anti-IL-17A antibody secukinumab, have emerged. These advancements enable more effective control of inflammation and slowing down of ossification, ultimately enhancing patients’ quality of life and prognosis (40). Moreover, diagnostic techniques have advanced, enabling earlier detection of AS and better disease management (41). Lastly, changes in people’s lifestyles and work habits over the past decade have led to alterations in the spectrum of diseases related to OA and AS. These changes may influence the bidirectional relationship between OA and AS. To further substantiate the reliability of our obtained results, we conducted a meta-analysis using a fixed-effects model, merging the outcomes of two datasets. The meta-analysis results endorse the initial conclusion from the MR analysis, affirming that AS functions as a risk factor for hand OA. The choice of a fixed-effects model was based on the absence of significant heterogeneity between the two results (42). As future research endeavors expand, there is a necessity to undertake further systematic reviews and meta-analyses to comprehensively evaluate and consolidate the evolving body of evidence in this domain.

Additionally, among the IVs extracted from GWAS data of AS from the FinnGen consortium, rs10807943 showed some correlation with both knee OA (*p* = 0.0419) and hip OA (*p* = 0.0407) outcomes. Although these *p*-values remained above the Bonferroni-corrected thresholds, we retained this IV to comprehensively assess the impact of each SNP. Leave-one-out sensitivity analysis indicated that excluding this SNP did not affect the outcomes, and the MR-PRESSO test did not detect horizontal pleiotropy. Notably, while no significant correlation was observed between the selected IVs and AS, the reverse MR analysis using hip OA as the exposure factor suggested the presence of horizontal pleiotropy. Despite negative results from all three models, including MR-Egger regression, which can adjust for pleiotropy, further evaluation of the direct correlation between hip OA and AS is warranted, in conjunction with clinical data.

The results of COLOC suggest that the *TNRC18* gene, located on chromosome 7, may be a pleiotropic gene associated with both AS and hand OA. *TNRC18* specifically recognizes histone H3K9me3 modification, mediating the silencing of endogenous retrotransposons (ERVs) to uphold the integrity and stability of the genome (43). Genetic factors play a crucial role in the pathogenesis of both AS and OA (23, 24). In this context, TNRC18 may influence the expression or regulation of homologous frame genes, thereby impacting the risk of developing AS and hand OA. Furthermore, COLOC analysis identified three loci on chromosome 6 with a colocalized relationship with both AS and hand OA. Hence, the relationship between AS and hand OA may involve intricate gene-gene interactions, and further exploration of specific mechanisms is warranted by subsequent researchers. Additionally, given that both AS and OA are complex multifactorial diseases, gene-environment interactions may influence the potential relationship between AS and OA. For example, previous MR studies have suggested a correlation between an increased BMI and a higher risk of OA (28). Therefore, the relationship between genetic variations related to AS and the risk of hand OA may be modulated by BMI, with a more pronounced susceptibility to OA genes evident when BMI is higher. Although our study did not yield significantly altered MR results upon incorporating BMI as an adjusting variable, it is essential for future research endeavors to delve further into the exploration of gene-environment interactions.

Our study possesses several strengths. Firstly, we systematically investigated the bidirectional relationship between AS and OA using the Mendelian randomization method, a topic relatively underexplored in previous research. Unlike traditional observational studies, MR studies leveraging genetic variation from the genome are less susceptible to confounding factors (44). Secondly, we rigorously applied Bonferroni correction for significance levels, ensuring that the *p*-values for positive results were all below the adjusted significance threshold. Furthermore, robust results were indicated by leave-one-out sensitivity analysis, reducing the risk of false positives. Lastly, we conducted our MR analysis meticulously, utilizing large-sample GWAS data from populations of European ancestry, with no overlap between exposure and outcome populations. In addition, we validated positive results by expanding the criteria for selecting IV and incorporating GWAS data from another cohort. By relaxing the IV selection criteria from a *p*-value of < 5 × 10^−8^ to < 1 × 10^−6^, we were able to identify more instrumental variables. This adjustment facilitated a more optimal balance between individual SNPs and the potential impact of horizontal pleiotropy on the results. Consequently, the amalgamation of SNP effects could be more precisely determined.

However, it’s crucial to acknowledge certain research limitations. Firstly, our study focused exclusively on individuals of European descent, and the incidence of AS varies across different ethnicities (45). Therefore, caution should be exercised when applying our findings to other racial groups. Furthermore, constrained by the methods employed in MR studies, complete resolution of potential confounding factors such as population heterogeneity and factors like age, gender, and dietary habits may not be achievable. Additionally, the influence of time in the onset process is overlooked. Moreover, the potential presence of horizontal pleiotropy in MR analysis could impact result accuracy. To mitigate this, we conducted comprehensive sensitivity analyses and validation processes. Lastly, our study is solely an observational study based on MR methods. While MR provides a relatively high level of evidence, it cannot establish a genuine causal relationship between ankylosing spondylitis and osteoarthritis. More experiments and clinical evidence are required to either support or validate these results.

Our study carries significant clinical implications. Firstly, our findings can assist clinicians in better understanding the heterogeneity between osteoarthritis in different joint sites and ankylosing spondylitis, along with potential variations in pathogenic mechanisms and influencing factors. Based on this information, we recommend that clinicians perform regular screening for osteoarthritis in ankylosing spondylitis patients, especially for hand joints, using appropriate radiographic or ultrasonographic methods, as well as assess the clinical symptoms and functional status of these patients. Moreover, we suggest that clinicians provide preventive measures for osteoarthritis in ankylosing spondylitis patients, such as physical therapy, exercise, weight control, and anti-inflammatory drugs, as well as individualized treatment options for hand joint osteoarthritis, such as analgesics, corticosteroid injections, or surgery, depending on the severity and progression of the disease. Furthermore, while our research suggests that there is no significant causal relationship between OA and AS at the genetic level, it remains crucial to consider the possibility of AS being misdiagnosed as OA (18). We advise that clinicians consider the possibility of ankylosing spondylitis in osteoarthritis patients, especially those with axial involvement, and perform appropriate diagnostic tests, such as HLA-B27 typing, sacroiliac joint imaging, or inflammatory markers, to confirm or exclude the diagnosis of ankylosing spondylitis.

Future researchers can draw upon the genetic-level correlation we have established between AS and OA to delve deeper into the causal mechanisms governing their relationship. Conducting clinical cohort studies or cross-sectional epidemiological observations using follow-up data from AS and OA patients, while meticulously accounting for potential confounding factors, can unveil the dynamic changes and influences between AS and OA. Moreover, the application of network meta-analysis could offer insights into the differential correlations between various types of OA and AS. This approach would not only validate the genetic-level correlation in broader populations but also contribute novel evidence to the field. Additionally, considering the potential correlation of AS and OA with patients’ lifestyles, future studies could use our preliminary research as a reference, collecting more detailed participant information such as dietary habits, physical activity levels, and other lifestyle factors. This would help further elucidate the role of these factors in the relationship between AS and OA.

## 5 Conclusions

Our MR analysis reveals that, among individuals of European ancestry, those with AS exhibit a lower risk of developing knee OA, while AS serves as a risk factor for hand OA. Furthermore, no significant correlation was found between AS and hip OA. However. reverse MR analysis suggests that OA does not significantly influence AS. COLOC analysis based on GWAS data from European populations identified four loci colocalized with AS and hand OA. This genetic-level insight challenges previous assumptions about the association between AS and the predilection for hip and knee involvement. Therefore, more experiment, epidemiological studies and clinical evidence is needed to support or validate our results and further elucidate the underlying mechanisms. Additionally, our study results offer new insights for genetic research on OA and AS.

## Data availability statement

The original contributions presented in the study are included in the article/**Supplementary Material**. Further inquiries can be directed to the corresponding authors.

## Ethics statement

Since all data were derived from previously published studies and public databases, no additional ethical approval was required. The studies were conducted in accordance with the local legislation and institutional requirements. The participants provided their written informed consent to participate in this study.

## Author contributions

YL: Conceptualization, Data curation, Formal Analysis, Investigation, Methodology, Project administration, Resources, Validation, Visualization, Writing – original draft, Writing – review & editing. DL: Data curation, Formal Analysis, Investigation, Methodology, Validation, Visualization, Writing – review & editing. HZ: Data curation, Formal Analysis, Writing – review & editing. HL: Data curation, Formal Analysis, Writing – review & editing. BY: Data curation, Writing – review & editing. YZ: Data curation, Writing – review & editing. HH: Data curation, Writing – review & editing. HS: Conceptualization, Investigation, Project administration, Resources, Supervision, Writing – review & editing.

## Supplementary material

Supplementary Figure 1 Leave-one-out sensitivity analysis. **(A)** AS on knee OA; **(B)** AS on hip OA; **(C)** AS on hand OA; **(D)** Knee OA on AS; **(E)** Hip OA on AS; **(F)** Hand OA on AS.

Supplementary Figure 2 Forest plot of the correlations between AS hand OA with different GWAS data sources.
