# Supplementary figures and images for: Causality between Ankylosing Spondylitis and osteoarthritis in European ancestry: a bidirectional Mendelian randomization study
Source: Front Immunol. 2024 Feb 6;15:1297454. doi: 10.3389/fimmu.2024.1297454 (PMC10876785; doi:10.3389/fimmu.2024.1297454)

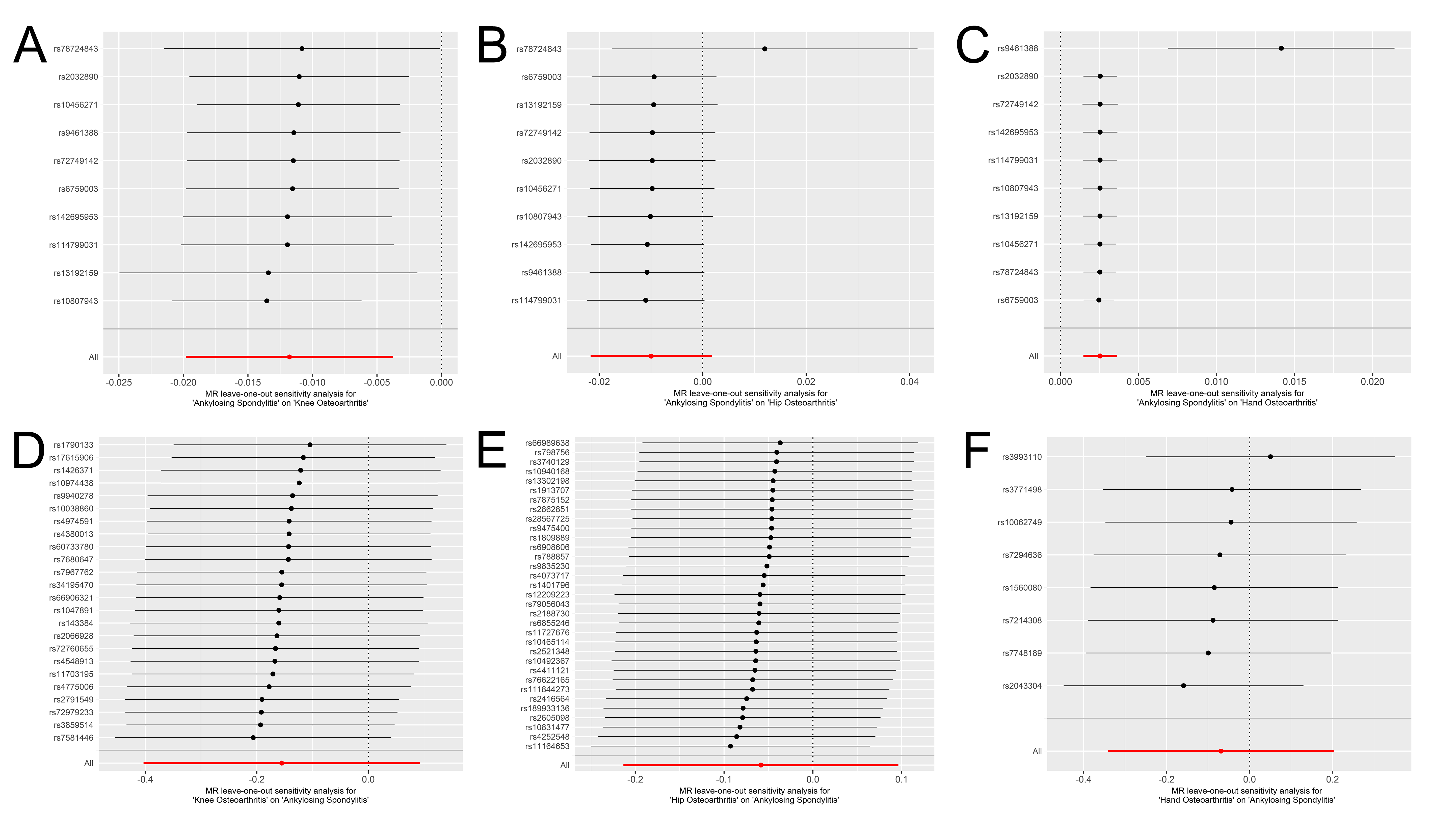

Supplement: Supplementary Figure 1 — Leave-one-out sensitivity analysis. (A) AS on knee OA; (B) AS on hip OA; (C) AS on hand OA; (D) Knee OA on AS; (E) Hip OA on AS; (F) Hand OA on AS. [file Image_1.tif]

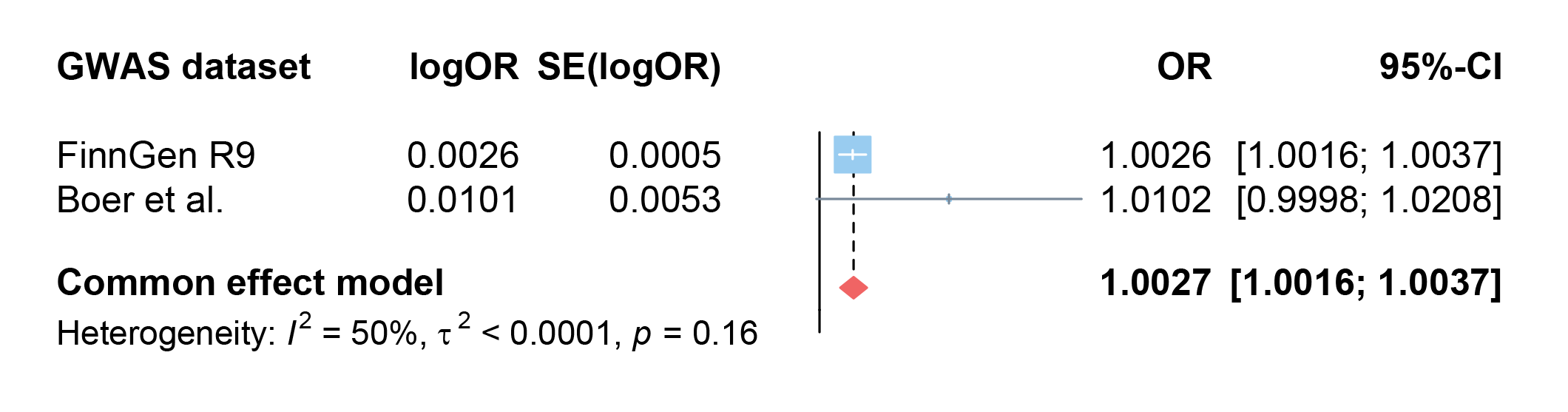

Supplement: Supplementary Figure 2 — Forest plot of the correlations between AS hand OA with different GWAS data sources. [file Image_2.tif]
