# Supplementary material for: Causality between Ankylosing Spondylitis and osteoarthritis in European ancestry: a bidirectional Mendelian randomization study
Source: Front Immunol. 2024 Feb 6;15:1297454. doi: 10.3389/fimmu.2024.1297454 (PMC10876785; doi:10.3389/fimmu.2024.1297454)
Supplement: Supplementary file 3 [file Table_1.docx]

**SUPPLYMENT TABLE 1.** GWAS data sources for MR analysis.

| Study | Phenotype | Year | Build | Dataset | Population | Sample size | No. of SNPs | No. of case | No. of control |
| --- | --- | --- | --- | --- | --- | --- | --- | --- | --- |
| FINNGEN R9 | AS | 2023 | HG19 | NA | European | 273,824 | 20,166,920 | 2,860 | 270,964 |
| Identification of multiple risk variants for ankylosing spondylitis through high-density genotyping of immune-related loci | AS | 2013 | HG19 | ebi-a-GCST005529  PMID: 23749187 | European | 22,647 | 99,962 | 9,069 | 13,578 |
| Deciphering osteoarthritis genetics across 826,690 individuals from 9 populations | OA | 2021 | HG19 | PMID: 34450027 | European  (major) | 826,690 | NA | 177,517 | 649,173 |
| UK Biobank | BMI | 2018 | HG19 | ukb-b-19953 | European | 461,460 | 9,851,867 | NA | NA |

AS: Ankylosing Spondylitis; OA: Osteoarthritis; BMI: Body mass index.
