# Supplementary material for: Causality between Ankylosing Spondylitis and osteoarthritis in European ancestry: a bidirectional Mendelian randomization study
Source: Front Immunol. 2024 Feb 6;15:1297454. doi: 10.3389/fimmu.2024.1297454 (PMC10876785; doi:10.3389/fimmu.2024.1297454)
Supplement: Supplementary file 4 [file Table_2.docx]

**SUPPLYMENT TABLE 2.** Instrumental variables for MR analysis GWAS-related information in exposure data and outcome data.

| SNP | A1 | A2 | EAF | Exposure GWAS | | | |  | | Outcome GWAS | | | | | |  |
| --- | --- | --- | --- | --- | --- | --- | --- | --- | --- | --- | --- | --- | --- | --- | --- | --- |
|  |  |  |  | Beta | SE | *p*-value | |  | | Beta | | SE | | *p*-value | |  |
| **a. AS as the exposure variable and knee OA as the outcome variable.** | | | | | | | | | | | | | | | |  |
| rs10456271 | T | C | 0.0118 | 0.4553 | 0.0676 | 1.61E-11 | |  | | 0.0213 | | 0.0120 | | 0.5418 | |  |
| rs10807943 | C | T | 0.1028 | -0.3737 | 0.0494 | 3.77E-14 | |  | | 0.0096 | | 0.0065 | | 0.0419 | |  |
| rs114799031 | T | A | 0.9747 | 1.2742 | 0.0364 | 8.28E-269 | |  | | -0.0614 | | 0.0921 | | 0.1327 | |  |
| rs13192159 | T | C | 0.1809 | 0.5337 | 0.0315 | 2.50E-64 | |  | | 0.0053 | | 0.0029 | | 0.5724 | |  |
| rs142695953 | A | C | 0.1391 | 0.2147 | 0.0330 | 7.38E-11 | |  | | -0.0176 | | 0.0196 | | 0.0934 | |  |
| rs2032890 | C | A | 0.6996 | -0.2432 | 0.0312 | 6.97E-15 | |  | | -0.0057 | | 0.0036 | | 0.4620 | |  |
| rs6759003 | C | T | 0.3609 | -0.1880 | 0.0277 | 1.12E-11 | |  | | -0.0095 | | 0.0088 | | 0.1944 | |  |
| rs72749142 | A | G | 0.1677 | -0.2353 | 0.0411 | 1.04E-08 | |  | | -0.0111 | | 0.0094 | | 0.2498 | |  |
| rs78724843 | A | C | 0.0334 | 0.6199 | 0.0538 | 9.54E-31 | |  | | 0.0083 | | 0.0037 | | 0.6822 | |  |
| rs9461388 | C | T | 0.9705 | -0.5790 | 0.0957 | 1.44E-09 | |  | | -0.0237 | | 0.0198 | | 0.2625 | |  |
| **b. AS as the exposure variable and hip OA as the outcome variable.** | | | | | | | | | | | | | | | |  |
| rs10456271 | T | C | 0.0118 | 0.4553 | 0.0676 | 1.61E-11 | |  | | 0.0595 | | 0.0574 | | 0.1676 | |  |
| rs10807943 | C | T | 0.1028 | -0.3737 | 0.0494 | 3.77E-14 | |  | | 0.0311 | | 0.0402 | | 0.0407 | |  |
| rs114799031 | T | A | 0.9747 | 1.2742 | 0.0364 | 8.28E-269 | |  | | -0.0362 | | 0.0296 | | 0.2769 | |  |
| rs13192159 | T | C | 0.1809 | 0.5337 | 0.0315 | 2.50E-64 | |  | | 0.0177 | | 0.0181 | | 0.1346 | |  |
| rs142695953 | A | C | 0.1391 | 0.2147 | 0.0330 | 7.38E-11 | |  | | -0.0102 | | 0.0066 | | 0.4423 | |  |
| rs2032890 | C | A | 0.6996 | -0.2432 | 0.0312 | 6.97E-15 | |  | | -0.0179 | | 0.0213 | | 0.0664 | |  |
| rs6759003 | C | T | 0.3609 | -0.1880 | 0.0277 | 1.12E-11 | |  | | -0.0099 | | 0.0079 | | 0.2933 | |  |
| rs72749142 | A | G | 0.1677 | -0.2353 | 0.0411 | 1.04E-08 | |  | | -0.0191 | | 0.0203 | | 0.1152 | |  |
| rs78724843 | A | C | 0.0334 | 0.6199 | 0.0538 | 9.54E-31 | |  | | 0.0083 | | 0.0032 | | 0.7537 | |  |
| rs9461388 | C | T | 0.9705 | -0.5790 | 0.0957 | 1.44E-09 | |  | | 0.0237 | | 0.0167 | | 0.3863 | |  |
| **c. AS as the exposure variable and hand OA as the outcome variable.** | | | | | | | | | | | | | | | |  |
| rs10456271 | T | C | 0.0118 | 0.4553 | 0.0676 | 1.61E-11 | |  | | -0.0202 | | 0.0082 | | 0.7309 | |  |
| rs10807943 | C | T | 0.1028 | -0.3737 | 0.0494 | 3.77E-14 | |  | | 0.0134 | | 0.0078 | | 0.5226 | |  |
| rs114799031 | T | A | 0.9747 | 1.2742 | 0.0364 | 8.28E-269 | |  | | -0.0416 | | 0.0294 | | 0.3828 | |  |
| rs13192159 | T | C | 0.1809 | 0.5337 | 0.0315 | 2.50E-64 | |  | | -0.0125 | | 0.0083 | | 0.4318 | |  |
| rs142695953 | A | C | 0.1391 | 0.2147 | 0.0330 | 7.38E-11 | |  | | -0.0173 | | 0.0134 | | 0.3176 | |  |
| rs2032890 | C | A | 0.6996 | -0.2432 | 0.0312 | 6.97E-15 | |  | | -0.0090 | | 0.0055 | | 0.4942 | |  |
| rs6759003 | C | T | 0.3609 | -0.1880 | 0.0277 | 1.12E-11 | |  | | 0.0022 | | 0.0006 | | 0.8589 | |  |
| rs72749142 | A | G | 0.1677 | -0.2353 | 0.0411 | 1.04E-08 | |  | | 0.0404 | | 0.0609 | | 0.1274 | |  |
| rs78724843 | A | C | 0.0334 | 0.6199 | 0.0538 | 9.54E-31 | |  | | -0.0135 | | 0.0057 | | 0.7117 | |  |
| rs9461388 | C | T | 0.9705 | -0.5790 | 0.0957 | 1.44E-09 | |  | | 0.0014 | | 0.0002 | | 0.9695 | |  |
| **d. Knee OA as the exposure variable and AS as the outcome variable.** | | | | | | | | | | | | | | | |  |
| rs10038860 | G | A | 0.274 | -0.0460 | 0.1447 | | 5.62E-09 | |  | | 0.0322 | | 0.0312 | | 0.3028 | |
| rs1047891 | C | A | 0.3082 | -0.0471 | 0.1418 | | 2.74E-08 | |  | | 0.0013 | | 0.0288 | | 0.9644 | |
| rs10974438 | A | C | 0.6434 | 0.0434 | 0.1370 | | 4.89E-09 | |  | | -0.0448 | | 0.0277 | | 0.1053 | |
| rs11703195 | G | T | 0.2356 | 0.0512 | 0.1637 | | 2.96E-09 | |  | | 0.0239 | | 0.0388 | | 0.5371 | |
| rs1426371 | G | A | 0.2707 | 0.0515 | 0.1697 | | 8.86E-10 | |  | | -0.0440 | | 0.0284 | | 0.1218 | |
| rs143384 | A | G | 0.5908 | 0.0722 | 0.3791 | | 1.01E-23 | |  | | -0.0079 | | 0.0271 | | 0.7709 | |
| rs17615906 | T | C | 0.8402 | -0.0523 | 0.1548 | | 4.88E-08 | |  | | 0.0920 | | 0.0406 | | 0.0233 | |
| rs1790133 | T | G | 0.2115 | -0.0687 | 0.2300 | | 4.49E-10 | |  | | 0.0701 | | 0.0347 | | 0.0436 | |
| rs2066928 | A | G | 0.4825 | -0.0412 | 0.1270 | | 1.20E-08 | |  | | -0.0038 | | 0.0269 | | 0.8892 | |
| rs2791549 | A | C | 0.2962 | 0.0457 | 0.1460 | | 3.06E-09 | |  | | 0.0370 | | 0.0290 | | 0.2013 | |
| rs34195470 | A | G | 0.4453 | -0.0523 | 0.2025 | | 3.13E-13 | |  | | 0.0080 | | 0.0270 | | 0.7661 | |
| rs3859514 | T | C | 0.3482 | 0.0435 | 0.1371 | | 5.12E-09 | |  | | 0.0464 | | 0.0299 | | 0.1205 | |
| rs4380013 | G | A | 0.1875 | -0.0562 | 0.1852 | | 8.73E-10 | |  | | 0.0412 | | 0.0445 | | 0.3540 | |
| rs4548913 | G | A | 0.6283 | 0.0510 | 0.1894 | | 3.15E-12 | |  | | 0.0041 | | 0.0274 | | 0.8806 | |
| rs4775006 | C | A | 0.4155 | -0.0464 | 0.1530 | | 8.55E-10 | |  | | -0.0167 | | 0.0270 | | 0.5369 | |
| rs4974591 | A | G | 0.3072 | -0.0421 | 0.1261 | | 3.26E-08 | |  | | 0.0245 | | 0.0287 | | 0.3927 | |
| rs60733780 | G | A | 0.3286 | 0.0433 | 0.1327 | | 1.46E-08 | |  | | -0.0243 | | 0.0298 | | 0.4154 | |
| rs66906321 | T | C | 0.1751 | -0.0565 | 0.1832 | | 1.71E-09 | |  | | 0.0034 | | 0.0363 | | 0.9254 | |
| rs72760655 | C | A | 0.3317 | -0.0489 | 0.1705 | | 7.25E-11 | |  | | -0.0051 | | 0.0290 | | 0.8599 | |
| rs72979233 | A | G | 0.7583 | -0.0496 | 0.1592 | | 2.54E-09 | |  | | -0.0438 | | 0.0321 | | 0.1721 | |
| rs7581446 | C | T | 0.4837 | 0.0565 | 0.1933 | | 1.71E-10 | |  | | 0.0343 | | 0.0269 | | 0.2017 | |
| rs7680647 | T | C | 0.6314 | -0.0442 | 0.1361 | | 1.24E-08 | |  | | 0.0205 | | 0.0272 | | 0.4518 | |
| rs7967762 | C | T | 0.1718 | -0.0570 | 0.1839 | | 2.09E-09 | |  | | 0.0091 | | 0.0317 | | 0.7742 | |
| rs9940278 | C | T | 0.4355 | -0.0580 | 0.2500 | | 3.19E-16 | |  | | 0.0253 | | 0.0270 | | 0.3500 | |
| **e. Hip OA as the exposure variable and AS as the outcome variable.** | | | | | | | | | | | | | | | |  |
| rs10465114 | G | A | 0.2203 | -0.0625 | 0.1941 | 9.04E-09 | |  | | -0.0104 | | 0.0325 | | 0.7494 | |  |
| rs10492367 | G | T | 0.1983 | -0.1147 | 0.6096 | 2.75E-24 | |  | | -0.0033 | | 0.0357 | | 0.9255 | |  |
| rs10831477 | T | G | 0.8137 | 0.0704 | 0.2302 | 1.20E-09 | |  | | 0.0700 | | 0.0371 | | 0.0590 | |  |
| rs10940168 | G | A | 0.3942 | 0.0534 | 0.1665 | 7.74E-09 | |  | | -0.0407 | | 0.0282 | | 0.1485 | |  |
| rs11164653 | T | C | 0.4131 | -0.0799 | 0.3666 | 2.77E-18 | |  | | -0.0440 | | 0.0270 | | 0.1029 | |  |
| rs111844273 | G | A | 0.0209 | -0.2317 | 0.8780 | 1.05E-12 | |  | | -0.2307 | | 0.1922 | | 0.2301 | |  |
| rs11727676 | T | C | 0.901 | -0.0849 | 0.2525 | 4.18E-08 | |  | | -0.0154 | | 0.0479 | | 0.7477 | |  |
| rs12209223 | C | A | 0.1117 | -0.1398 | 0.7135 | 1.88E-22 | |  | | 0.0065 | | 0.0391 | | 0.8685 | |  |
| rs13302198 | C | T | 0.6445 | -0.0544 | 0.1618 | 4.14E-08 | |  | | 0.0351 | | 0.0280 | | 0.2094 | |  |
| rs1401796 | C | A | 0.5129 | 0.0583 | 0.2003 | 1.43E-10 | |  | | -0.0086 | | 0.0272 | | 0.7525 | |  |
| rs1809889 | T | C | 0.2806 | 0.0596 | 0.1896 | 3.58E-09 | |  | | -0.0294 | | 0.0292 | | 0.3143 | |  |
| rs189933136 | T | C | 0.8413 | 0.1140 | 0.4716 | 5.33E-15 | |  | | 0.0439 | | 0.0427 | | 0.3040 | |  |
| rs1913707 | A | G | 0.6043 | 0.0677 | 0.2646 | 1.82E-13 | |  | | -0.0274 | | 0.0273 | | 0.3148 | |  |
| rs2188730 | G | A | 0.1505 | -0.0722 | 0.2235 | 1.01E-08 | |  | | -0.0033 | | 0.0402 | | 0.9337 | |  |
| rs2416564 | C | T | 0.5981 | 0.0734 | 0.3112 | 1.00E-15 | |  | | 0.0229 | | 0.0281 | | 0.4160 | |  |
| rs2521348 | C | T | 0.3908 | -0.0555 | 0.1803 | 1.56E-09 | |  | | -0.0089 | | 0.0273 | | 0.7438 | |  |
| rs2605098 | A | G | 0.3368 | 0.0651 | 0.2382 | 6.83E-12 | |  | | 0.0357 | | 0.0280 | | 0.2024 | |  |
| rs28567725 | T | C | 0.5732 | -0.0546 | 0.1770 | 1.72E-09 | |  | | 0.0300 | | 0.0272 | | 0.2699 | |  |
| rs2862851 | T | C | 0.4653 | 0.0655 | 0.2527 | 3.86E-13 | |  | | -0.0261 | | 0.0271 | | 0.3356 | |  |
| rs3740129 | G | A | 0.4505 | -0.0547 | 0.1770 | 1.84E-09 | |  | | 0.0451 | | 0.0284 | | 0.1126 | |  |
| rs4073717 | G | T | 0.2013 | 0.0672 | 0.2157 | 2.54E-09 | |  | | -0.0143 | | 0.0342 | | 0.6760 | |  |
| rs4252548 | C | T | 0.0276 | -0.2254 | 0.9446 | 2.24E-15 | |  | | -0.1219 | | 0.0842 | | 0.1478 | |  |
| rs4411121 | C | T | 0.3137 | -0.0648 | 0.2317 | 2.16E-11 | |  | | -0.0108 | | 0.0296 | | 0.7160 | |  |
| rs66989638 | G | A | 0.1326 | -0.0789 | 0.2492 | 4.79E-09 | |  | | 0.0572 | | 0.0345 | | 0.0978 | |  |
| rs6855246 | A | G | 0.9277 | -0.1087 | 0.3387 | 7.94E-09 | |  | | -0.0367 | | 0.1098 | | 0.7386 | |  |
| rs6908606 | A | G | 0.711 | -0.0688 | 0.2545 | 3.86E-12 | |  | | 0.0232 | | 0.0292 | | 0.4264 | |  |
| rs76622165 | C | T | 0.2225 | -0.0608 | 0.1817 | 3.56E-08 | |  | | -0.0206 | | 0.0315 | | 0.5119 | |  |
| rs7875152 | C | A | 0.1408 | 0.0935 | 0.3542 | 1.07E-12 | |  | | -0.0360 | | 0.0376 | | 0.3377 | |  |
| rs788857 | A | G | 0.7004 | -0.0544 | 0.1615 | 4.42E-08 | |  | | 0.0244 | | 0.0278 | | 0.3789 | |  |
| rs79056043 | A | G | 0.9367 | -0.1136 | 0.3669 | 1.99E-09 | |  | | 0.0017 | | 0.0576 | | 0.9767 | |  |
| rs798756 | T | C | 0.1936 | -0.0683 | 0.2199 | 2.24E-09 | |  | | 0.0484 | | 0.0325 | | 0.1361 | |  |
| rs9475400 | C | T | 0.0987 | -0.1084 | 0.4128 | 8.03E-13 | |  | | 0.0475 | | 0.0481 | | 0.3241 | |  |
| rs9835230 | G | A | 0.2433 | -0.0638 | 0.2081 | 1.34E-09 | |  | | 0.0222 | | 0.0329 | | 0.4983 | |  |
| **f. Hand OA as the exposure variable and AS as the outcome variable.** | | | | | | | | | | | | | | | |  |
| rs10062749 | G | T | 0.2691 | -0.0814 | 0.2627 | 2.04E-09 | |  | | 0.0192 | | 0.0317 | | 0.5455 | |  |
| rs1560080 | G | A | 0.8252 | 0.0916 | 0.2840 | 9.61E-09 | |  | | 0.009- | | 0.0426 | | 0.8334 | |  |
| rs2043304 | T | C | 0.3287 | 0.0728 | 0.2217 | 1.84E-08 | |  | | 0.0458 | | 0.0299 | | 0.1249 | |  |
| rs3771498 | C | T | 0.5206 | 0.0794 | 0.2772 | 6.81E-11 | |  | | -0.0159 | | 0.0269 | | 0.5540 | |  |
| rs3993110 | A | C | 0.6075 | 0.0823 | 0.2910 | 3.75E-11 | |  | | -0.0516 | | 0.0272 | | 0.0580 | |  |
| rs7214308 | C | T | 0.7303 | -0.0748 | 0.2220 | 4.47E-08 | |  | | -0.0065 | | 0.0313 | | 0.8348 | |  |
| rs7294636 | G | A | 0.3716 | -0.0706 | 0.2159 | 1.59E-08 | |  | | 0.0034 | | 0.0298 | | 0.9108 | |  |
| rs7748189 | G | A | 0.7315 | -0.0799 | 0.2507 | 6.13E-09 | |  | | -0.0148 | | 0.0338 | | 0.6616 | |  |

A1: Effect allele; A2: Other allele; SE: Standard error.
