# Supplementary material for: Causality between Ankylosing Spondylitis and osteoarthritis in European ancestry: a bidirectional Mendelian randomization study
Source: Front Immunol. 2024 Feb 6;15:1297454. doi: 10.3389/fimmu.2024.1297454 (PMC10876785; doi:10.3389/fimmu.2024.1297454)
Supplement: Supplementary file 5 [file Table_3.docx]

**SUPPLYMENT TABLE 3.** Combined results from other Mendelian randomization models.

| Exposure | Outcome | Model 1 | |  | Model 2 | |  | Model 3 | |
| --- | --- | --- | --- | --- | --- | --- | --- | --- | --- |
|  |  | OR | 95% CI |  | OR | 95% CI |  | OR | 95% CI |
| AS | Knee OA | 0.9817 | 0.9671-0.9966 |  | 0.9883 | 0.9671-0.9966 |  | 0.9882 | 0.9811-0.9954 |
| AS | Hip OA | 1.0236 | 0.9897-1.0585 |  | 1.0109 | 1.0001-1.0211 |  | 1.0100 | 1.0006-1.0195 |
| AS | Hand OA | 1.0284 | 1.0118-1.0453 |  | 1.0025 | 1.0014-1.0036 |  | 1.0028 | 1.0015-1.0041 |
| Knee OA | AS | 0.8769 | 0.7441-1.0333 |  | 0.9635 | 0.8038-1.1550 |  | 0.5699 | 0.3262-0.9953 |
| Hip OA | AS | 0.9548 | 0.8644-1.0547 |  | 1.0056 | 0.8846-1.1432 |  | 0.7571 | 0.5043-1.1368 |
| Hand OA | AS | 1.0202 | 0.9017-1.1543 |  | 0.8850 | 0.6394-1.2250 |  | 0.7020 | 0.3405-1.4473 |

*Note.* Model 1: simple median; Model 2: penalized weighted median; Model 3: maximum likelihood.
