# Supplementary material for: Causality between Ankylosing Spondylitis and osteoarthritis in European ancestry: a bidirectional Mendelian randomization study
Source: Front Immunol. 2024 Feb 6;15:1297454. doi: 10.3389/fimmu.2024.1297454 (PMC10876785; doi:10.3389/fimmu.2024.1297454)
Supplement: Supplementary file 6 [file Table_4.docx]

**SUPPLYMENT TABLE 4.** Gene colocalization analysis of AS and OA.

| Outcome | SNP | Position | Alleles | Nearest Gene | Distance | PP4 |
| --- | --- | --- | --- | --- | --- | --- |
| AS-hand OA | rs74707996 | 6:23732205 | G>T | *NRSN1* | >100bp | 0.9999 |
| AS-hand OA | rs75240935 | 6:25890886 | G>T | *SLC17A3* | >100bp | 0.9999 |
| AS-hand OA | rs181468789 | 6:28013596 | G>A | *OR2B6* | >100bp | 0.9484 |
| AS-hand OA | rs748670681 | 7:5397122 | C>T | *TNRC18* | 0bp | 1 |
